# Association of regular physical activity with total and cause-specific mortality among middle-aged and older Chinese: a prospective cohort study

**DOI:** 10.1038/srep39939

**Published:** 2017-01-04

**Authors:** Yun Zhou, Runbo Zhang, Yuewei Liu, Yanjun Guo, Dongming Wang, Meian He, Jing Yuan, Yuan Liang, Xiaomin Zhang, Youjie Wang, Huan Guo, Sheng Wei, Xiaoping Miao, Ping Yao, Tangchun Wu, Weihong Chen

**Affiliations:** 1 Department of Occupational and Environmental Health, School of Public Health, Tongji Medical College, Huazhong University of Science and Technology, Wuhan, China; 2 Key Laboratory of Environment and Health in Ministry of Education & Ministry of Environmental Protection, and State Key Laboratory of Environmental Health (Incubating), School of Public Health, Tongji Medical College, Huazhong University of Science and Technology, Wuhan, China; 3 Hubei Provincial Key Laboratory for Applied Toxicology, Hubei Provincial Center for Disease Control and Prevention, Wuhan, Hubei 430079, China; 4 Department of Epidemiology and Biostatistics, School of Public Health, Tongji Medical College, Huazhong University of Science and Technology, Wuhan 430030, Hubei, China

## Abstract

Association between physical activity and mortality has rarely been investigated among the Chinese population. Furthermore, the most appropriate amount of physical activity for longevity benefits remains unclear. We used data from the Dongfeng-Tongji cohort, including 24,606 middle-aged and older retired adults in 2008 and followed to 2013, to quantify linear and non-linear dose-response relationships between regular physical activity and mortality risks by Cox proportional hazards model. Compared with participants who did not engage in regular physical activity, those performing regular physical activity had significantly 46%, 56%, and 49% decreased risks of mortality from all causes, circulatory, and respiratory diseases, respectively. Each one-SD increase in regular physical activity was associated with 32% decrease of respiratory disease mortality. There were significant nonlinear dose-response associations between regular physical activity and mortality from all causes and circulatory diseases. Mortality risks decreased monotonically with increased regular physical activity amount, and appeared to reach a threshold at around 100 MET-hours/week. More mortality benefits were found among non-smokers than that among current and former smokers. Our results suggest that middle-aged and older Chinese adults can achieve mortality benefits from regular physical activity at the WHO recommended minimum, and the benefit threshold appears at approximately 100 MET hours/week.

Physical activity has been associated with decreased risk of mortality from all causes, cardiovascular diseases and cancers^1^,^2^,^3^. According to data from the World Health Organization (WHO), lack of physical activity has been identified as the fourth leading risk factor for death globally^4^. It was estimated that 3.2 million deaths were attributed to insufficient physical activity every year^5^. WHO recommends that adults do at least 150 minutes of moderate intensity (3 to <6 metabolic equivalent tasks [MET, a physiological measure, can express the energy cost of physical activities. A MET equals one kcal per hour for per kilogram of bodyweight]) or 75 minutes of vigorous intensity (≥6 METs) aerobic physical activity throughout a week (≥7.5 MET hours/week) or an equivalent combination of moderate and vigorous intensity activity^6^. In China, over 66% of the general population participated in physical activity beyond the WHO recommended minimum^7^. Currently, no guidelines for physical activity are available in China, and whether Chinese adults can benefit from physical activity according to the WHO recommendation remains unclear.

Cumulative evidence has shown that physical activity can decrease mortality; however, the health benefits among elderly remain fairly sparse. While the health benefits appear greater among older populations than younger adults^8^, adverse effects of physical activity are also found among the elderly population as it can be of trigger of sudden deaths^9^,^10^,^11^. Therefore, it is of importance and interest to identify if there is a threshold for mortality risk associated with physical activity especially among elderly. To date, only one study among the US general populations reported that an upper threshold for mortality benefits occurred at 22.5 to 40.0 MET-hours/week, though leisure-time physical activity of more than 40 MET-hours/week did not appear to be associated with elevated mortality risk^12^. However, this study did not account for non-leisure time activities especially occupational physical activity, which might result in exposure misclassification. A clearer separation of occupational and non-occupational physical activity benefits is needed to be addressed.

In this study, we investigated the associations between regular physical activity and risks from total and cause-specific mortality in the Dongfeng-Tongji (DFTJ) cohort of 24 606 middle-aged and older retired Chinese. Both linear and nonlinear dose-response analyses were conducted to quantify the associations and identify potential thresholds for mortality risk associated with regular physical activity.

## Results

Table 1 summaries selected characteristics of the study participants by regular physical activity. Over 44.3% of the participants were males. Mean age of all the participants was 63 ranging from 45 to 95 years. Approximately 95% of the participants performed physical activity less than 75 MET-hours/week, including 2862 participants who reported no regular physical activity. There are 82.2% of subjects performing equal to or more than the WHO recommended minimum (7.5 MET-hours/week). Among all the participants, 41.2% were overweight (28 > BMI ≥ 25) or obese (BMI ≥ 28), 2.6% were underweight, and 2.5% were without data on BMI. Current and former smokers accounted for 29.5% of all participants, while current and former drinker accounted for 26.6%.

Mortality rates by regular physical activity is presented in Table 2. There were 125,591 person-years in total. The overall mortality was 10.0 per 1000 person-years. The identified 1267 deaths included 436 (mortality rate: 3.5 per 1000 person-years) deaths from neoplasms, 458 (3.6) from circulatory diseases, 84 (0.7) from respiratory diseases, 37 (0.3) from digestive diseases, 46 (0.4) from external causes of morbidity and mortality.

Table 3 shows results of the dose-response analyses for regular physical activity and mortality risk. Compared with no regular physical activity, participants reporting regular physical activity show a significant 46%, 56%, and 49% decrease of mortality from all causes, circulatory diseases and respiratory diseases, respectively. Each one-SD increase (24.3 MET-hours/week) in regular physical activity was associated with 22%, 30%, and 32% decrease of mortality from all causes, circulatory diseases and respiratory diseases, respectively. The categorical analyses also showed similar results with significant decreasing Hazard Ratio (HR) trends for categories of regular physical activity in relation to total and cause-specific mortality. Participants performing regular physical activity but less than 7.5 MET-hours/week (the WHO recommended minimum) were also significantly associated with reductions of mortality risk from total and circulatory diseases (HR: 0.69 [0.54–0.88] and 0.65 [0.44–0.95] respectively). No significant associations were observed between lower mortality risks from neoplasms and respiratory diseases and increasing regular physical activity amount until 37.5 to 75 MET-hours/week. The results also showed that total and neoplasms mortality risks decreased monotonically with increasing regular physical activity amount. For circulatory diseases and respiratory diseases, the mortality risks decreased and appeared to reach a threshold among participants performing 35.7 to 75 MET-hours/week regular physical activity. The results of sensitivity analysis among those who are in normal retirement age (more than 60 years old for male and 50 for female in China) are similar to those in the whole participants (data not shown).

**Figure 1:**
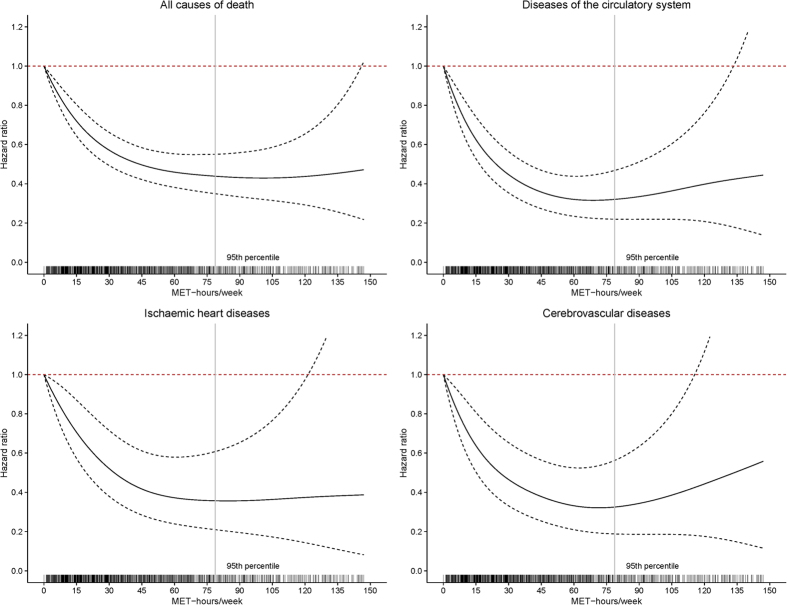
Estimated hazard ratios of mortality associated with regular physical activity by non-linear spline models (N = 24,606). Abbreviations: MET, metabolic equivalent. SD, standard deviation. The causes of death were coded according to the 10^th^ version of the International Classification of Diseases (ICD-10). Hazard ratios were estimated by using the Cox proportional hazards model, with adjustment for sex, age, BMI, marriage, education, smoking status, and drinking status at baseline. The horizontal dotted red line in each panel represents a HR of 1.0. Solid lines represent HRs, with dotted lines indicating the 95% CI.

We further examined the nonlinear dose-response associations for regular physical activity and risks of mortality by the penalized spline models. There were significant nonlinear dose-response associations for regular physical activity and risks of mortality from all causes, circulatory diseases, ischaemic heart diseases and cerebrovascular diseases (all *p* values for nonlinear trend <0.05) (Fig. 1). The results showed that total mortality risk decreased monotonically, and appeared to reach a threshold at around 100 MET-hours/week. No significant nonlinear associations were observed between regular physical activity amount and risks of mortality from neoplasms and respiratory diseases.

As shown in Table 4, stratified analyses gave similar dose-response associations between regular physical activity and total mortality risk by age, BMI, marriage status, education, and drinking status; however, we observed different associations modified by smoking status (*p* effect modification = 0.04). Regular physical activity appeared to decrease total mortality risk more among non-smokers than ever smokers (including current and former smokers).

## Discussion

In this prospective cohort study of 24 606 middle-aged and older Chinese retired adults, we observed significant dose-response associations between regular physical activity and decreased risks of mortality from all diseases, neoplasms, circulatory diseases and respiratory diseases. The association can be modified by smoking. We also found Chinese middle-aged and older adults can achieve mortality benefits from regular physical activity at the WHO recommended minimum, and the benefit threshold appears at approximately 100 MET hours/week.

Many studies have demonstrated that increased physical activity was associated with total mortality risk decline. A dose-response meta-analysis of 80 cohort studies reported that physical activity at the highest level can lower 35% risk of total mortality when compared with the lowest level^8^. A pooled analysis from six studies in United States and Europe to conduct the dose-response relationship between physical activity and mortality, reporting a lower risk between 20 and 39% of total mortality among those with physical activity compared with those reporting no physical activity^12^. Our findings showed that participants with regular physical activity were associated with a 31 to 61% lower risk of total mortality when compared with those who did not engage in regular physical activity, which are higher than the results from Arem *et al*. and Wen *et al*.^12^,^13^. An important reason is the different measurement of physical activity amount between the studies. According to the WHO recommendation, physical activity includes leisure-time physical activity, transportation, occupational, household chores, exercise, and community activities^14^. Arem *et al*. and Wen *et al*. assessed the effects of leisure-time physical activity on mortality, while in this study we focused on regular physical activities not only leisure-time physical activity, but also transportation and exercise. Another possible reason for more mortality benefits in regular physical activity in this study is that types and intensity of physical activity among the Chinese population are different from adults in some Western studies. Chinese people prefer moderate intensity physical activities such as walking, biking, and tai chi to vigorous intensity activities^15^. A few studies have assessed the effects of physical activity intensity independently on mortality risk, but the results are inconsistent. A study among late middle aged adults showed that moderate physical activity was associated with a 33% decreased risk of mortality, while no significant association was observed between vigorous activity and mortality^16^. In contrast, several studies have concluded that vigorous intensity physical activity may have a greater benefit for reducing risk of mortality than moderate intensity among adults aged between 28 and 86^8^,^17^. Age is another factor affecting the association between physical activity and mortality. A larger reduction in mortality tends to be observed among older than younger adults^8^,^18^. In our study, 62% of the participants were aged over 60. Stratified analyses suggested that each one-SD increase in regular physical activity was associated with a 16–28% reduction of total mortality among adults aged over 60, whilst there was 19% reduction among those aged under 60 in this study. This may have been due to retirement transition in the elderly as physical activity patterns and amount can alter after retirement. All the subjects enrolled in this study are all in retirement. Chinese older people who are retired do not go to work, therefore they will spend more time on mild or moderate physical activity such as walking, dancing, or playing tai chi, compared to those are working. Barnett *et al*. reported that the amount of exercise and leisure-time physical activity increases after the retirement transition^19^. Similarly, a recent longitudinal study has confirmed this and observed that the amount and intensity of physical activity could change by the type of transition out of full-time employment among middle-aged and older adults^20^. All participants enrolled in this study were retired workers, who have no occupational physical activity and may spend more time on regular non-occupational physical activity, gaining more mortality benefits from physical activity than those in other studies.

Physical activity is believed to help reduce cancer risk, but the results are inconsistent among studies in the elderly^1^,^12^,^21^,^22^. A cohort study of Japanese elderly adults reported no clear associations between an overall index of physical activity and cancer mortality, although a moderate reduction of risk was observed^22^; while Wu *et al*. found frequency of physical activity were associated with a lower cancer mortality^21^. In our study, we did not observe a significant association between neoplasms mortality and physical activity amount among lower than 37.5 MET-hour/week groups. A remarkable reduction of neoplasms mortality was observed when participants performed more than 37.5 MET-hours/week physical activity. A possible reason is the relatively small sample size for neoplasms, because the association between regular physical activity and neoplasm mortality is likely to be weak and difficult to detect. Further studies with larger sample sizes are needed to examine possible effects of physical activity on cancer mortality among elderly.

Our findings show that performing regular physical activity is an efficient way to reduce the risk of dying from diseases of the circulatory system, which are similar to previous studies^23^,^24^. Santulli *et al*. conducted a review on the benefits of moderate-intensity physical activity and found positive associations between physical training and aging^25^. According to Shiroma *et al*., the most active individuals have a 30 to 40% risk reduction of CHD and CVD, compared with the least active^26^. In our study, we found a 56% reduction of mortality from circulatory system diseases. It suggests that Chinese adults can benefit more from physical activity. Race may influence the dose-response association between risk of mortality from heart diseases and physical activity amount. Nonblack individuals more likely benefit from physical activity than blacks, but the association among Asians is still not clear because of rarely examined^27^.

We also noted that smoking was a potential effect modifier of the association. A greater reduction of mortality risk is associated with increased regular physical activity amount among non-smokers compared with those among smokers, which is similar to a cross-sectional and longitudinal analyses among Swedish adults^28^. Both smoking and physical inactivity can increase mortality risk independently. In this study, smokers typically participated in less physical activities compared with non-smokers, which might lead to greater reduction of total mortality among non-smokers. However, a study among 40–60 year-old employees in Finland reported that physical activity amount can lower mortality risk among smokers, but no association was found among non-smokers^29^.

Our findings show that regular physical activity can decrease mortality risk among middle-aged and older Chinese adults, suggesting that intervention strategies to promote regular physical activity should be a major health priority. Until now, there are no guidelines for physical activity levels in the mainland of China. In the present study, the regular physical activity levels lower than the WHO recommended minimum (0.1 to 7.4 MET-hours/week) are observed to be associated with a 31% reduction in risk of total mortality, which is higher than that from the report of the Physical Activity Guidelines Advisory Committee^30^. Furthermore, an increase in the amount of physical activity is associated with an additional reduction of total mortality risk at smaller magnitudes, but it seems that 7.5 MET-hours/week of physical activity is effective in reducing mortality and should be achievable among middle-aged and older retired adults in China. We also noted that the benefit threshold of regular physical activity appears to be 100 MET-hours/week. There are few studies which focus on the upper threshold of physical activity^12^, however, one study which pooled data from 6 studies in the National Cancer Institute Cohort Consortium reported that a threshold for mortality benefit occurred at 3–5 times the WHO recommendation. The study reported a reduction of mortality risk at the highest physical activity of equal or more than 75 MET-hours/week, but there was no detail on mortality benefit for physical activity beyond 75 MET-hours/week. Our results confirm and extend the previous study to calculate the upper threshold by using both categorical and non-linear models. We still observed a reduced risk of total mortality at more than 5 times the recommended minimum and a higher upper threshold appeared at 100 MET-hours/week^12^. This indicates that people can achieve more mortality benefits through performing more regular physical activity up to 100 MET-hours/week. These findings have important public health implications on future guidelines of regular physical activity, especially in China.

There are some strengths of this study. First, all participants enrolled in this study are retired workers, eliminating potential confounding by occupational physical activity. Second, we had a wide range of regular physical activity amount, which enabled us to examine the upper threshold of mortality benefits. Finally, we quantified the dose-response relationship between regular physical activity and mortality by both linear and non-linear analyses, and the results from the different models are consistent. Meanwhile, nonlinear analyses represent a detail dose-response association between regular physical activity and mortality, especially at high level of physical activity.

One limitation in our study is that the relatively short follow-up time. All the information about physical activity is self-reported. Recall bias on duration of regular physical activity, especially in earlier years may exist. Moreover, we did not measure the accurate intensity of certain type of regular physical activity. Instead, we assessed the physical activity intensity by types of physical activity according to MET equivalents of common physical activities classification. Furthermore, in this study, we included participants in early retirement. According to the State Council Provisional Regulations on Retirement and Resignation of Workers in China, males who are reaching the age of 50, females 45, with a seniority of ten years of continuous service, certified in hospital and confirmed by the labor appraisal committee as being completely incapacitated should retire. Younger subjects in our study may cause bias on the relationship. However, we excluded those suffering severe diseases which may affect physical activities in the present study. Meanwhile, we conducted a sensitive analysis to quantify the association between mortality risks and regular physical activity among those a participants retired in the normal age (more than 60 years old for male and 50 for female), and the results are similar to those among whole subjects. Another limitation is that we did not examine any effects of types, intensity, duration, or frequency of physical activity independently, although we considered all of them when estimating the physical activity amount. The types and intensity of physical activity among Chinese adults maybe different from individuals in Western countries. Further studies are needed to investigate the associations between types or intensity of physical activity and mortality risk among Chinese adults.

In conclusion, regular physical activity is negatively associated with total and cause-specific mortality risks, and the benefit threshold from regular physical activity appears at approximately 100 MET hours/week among middle-aged and older Chinese retired adults. Non-smokers might gain more health benefits from regular physical activity. Regular physical activity should therefore be encouraged to promote positive health outcomes in China.

## Methods

### Study design, setting and participants

This study included 24 606 participants from the DFTJ cohort, which has been described elsewhere^31^. In brief, the cohort included 27 009 residents in retirement and from different communities in Shiyan city, China. It was established and collected for baseline information between September 2008 and June 2010, and then followed up until October 31, 2013. A total of 25 978 participants (96.2%) completed the follow-up, yielding in 125 591 person-years with a median of 5.1 years follow-up. Trained investigators used questionnaires to collect information on demographics, education, marriage status, smoking, alcohol consumption, regular physical activity, and vital status. By excluding 479 participants with a diagnosis of malignant neoplasms and 893 participants without information on regular physical activity, 24 606 participants were included in the final analysis.

### Ethics Statement

The research protocol was approved by the Ethics and Human Subject Committee of Tongji Medical College, Huazhong University of Science and Technology, P.R. China. The methods were carried out in accordance with the relevant guidelines. All participants enrolled gave written informed consent for participation.

### Exposure assessment

We collected baseline self-reported information on regular physical activity from each participant through the questionnaire by asking five questions: “Did you do regular physical activity in the past six month, which lasts at least 20 minutes each time?”, “Which type of physical activity did you do? (Including leisure-time physical activity, transportation and exercises)”, “How many times did you do on regular physical activity each week?” “How long did you spend on physical activity each time?”, “How many years have you done regular physical activity?” We considered 9 types of regular physical activity, including walking, biking, dancing, tai chi, doing exercise in gym, playing ball games, jogging, swimming, and climbing. For each participant, energy expended by each type of physical activity was calculated by multiplying estimated metabolic equivalent (MET) value by the duration in hours/week. The estimated MET hours/week for regular physical activities were 3 for walking, 4 for biking, 4.5 for tai chi, 7.5 for jogging or swimming, 5 for dancing, 4.5 for climbing, 6 for playing ball games or doing exercise in gym^32^,^33^. We then summed across all types of regular physical activities for each subject to estimate the overall energy expenditure in MET hours/week.

### Outcomes

Each subject has a unique medical insurance card number in the medical insurance system. We used this system to track each subject’s vital status, and obtained underlying cause of death for each deceased. The 10^th^ version of the International Classification of Diseases (ICD-10) was used to code the causes of death. In this study, we divided causes of death into those resulting from all causes (ICD-10: A00-Y98), and specific diseases including Neoplasms (C00-C97), diseases of the circulatory system (I00-I99), and diseases of the respiratory system (J00-J99).

### Statistical analysis

We conducted quantitative dose-response analyses using the Cox proportional hazards model. HR and 95% confidence intervals (CIs) for mortality risk with regular physical activity were estimated, with adjustment for sex, age, BMI, marriage status, education, smoking status, and drinking status. By including regular physical activity as a dichotomized variable (yes/no), we estimated the HR for regular physical activity in relation to mortality risk in comparison with no regular physical activity. We also estimated the HR associated with each one-standardized deviation (SD) increase in regular physical activity, by including regular physical activity as a continuous variable in the model. To examine shape of the dose-response relationship, we conducted categorical analyses by dividing regular physical activity into six categories, including no regular physical activity (0), 0.1 to <7.5, 7.5 to <22.5, 22.5 to <37.5, 37.5 to <75, and ≥75 MET-hours/week. The cutpoints of these categories were 1, 3, 5, and 10 times the recommended minimum of WHO physical activity guidelines (7.5 MET-hours/week), respectively. In addition, we examined nonlinearity of the dose-response relationships by integrating penalized splines in the Cox proportional hazards model. Furthermore, we conducted a sensitivity analysis to explore the potential effect of physical activity on mortality among those who were in early retirement.

We conducted stratified analyses by using Cox proportional hazards models to investigate whether covariates modified the association between regular physical activity and total mortality risk, Effect modification of each stratification variable was performed by likelihood ratio tests by comparing two nested multivariate models with and without an interaction term of regular physical activity and the stratification variable. A *p* value larger than 0.05 was considered no significant effect modification. We conducted all data analyses using R 3.2.3 software. All *p* values were 2-sided.

## Additional Information

**How to cite this article**: Zhou, Y. *et al*. Association of regular physical activity with total and cause-specific mortality among middle-aged and older Chinese: a prospective cohort study. *Sci. Rep.* **7**, 39939; doi: 10.1038/srep39939 (2017).

**Publisher's note:** Springer Nature remains neutral with regard to jurisdictional claims in published maps and institutional affiliations.

## Figures and Tables

**Table 1:** Characteristics of the study participants by regular physical activity in MET-hours/week.

Characteristic	Overall	Regular physical activity in MET-hours/week					
		0	0.1 to <7.5	7.5 to <22.5	22.5 to <37.5	37.5 to <75.0	≥75
Number of participants	24 606	2862	1521	8707	5110	4995	1411
Male sex (%)	10 912 (44.3)	1164 (40.7)	585 (38.5)	3624 (41.6)	2316 (45.3)	2469 (49.4)	754 (53.4)
Age, years (%)							
≤60	9353 (38)	1276 (44.6)	633 (41.6)	3231 (37.1)	1951 (38.2)	1783 (35.7)	479 (33.9)
61–70	11 066 (45)	1112 (38.9)	593 (39)	3833 (44)	2368 (46.3)	2423 (48.5)	737 (52.2)
>70	4187 (17)	474 (16.6)	295 (19.4)	1643 (18.9)	791 (15.5)	789 (15.8)	195 (13.8)
BMI, kg/m^2^ (%)							
<18.5	641 (2.6)	85 (3)	53 (3.5)	227 (2.6)	122 (2.4)	133 (2.7)	21 (1.5)
18.5–24.9	13 208 (53.7)	1461 (51)	813 (53.5)	4506 (51.8)	2853 (55.8)	2768 (55.4)	807 (57.2)
25–27.9	6668 (27.1)	747 (26.1)	382 (25.1)	2404 (27.6)	1375 (26.9)	1371 (27.4)	389 (27.6)
≥28	3466 (14.1)	479 (16.7)	218 (14.3)	1329 (15.3)	651 (12.7)	618 (12.4)	171 (12.1)
Married (%)	22 070 (89.7)	2542 (88.8)	1339 (88)	7771 (89.3)	4629 (90.6)	4498 (90.1)	1291 (91.5)
Lower education* (%)	16 101 (65.4)	1964 (68.6)	964 (63.4)	5592 (64.2)	3294 (64.5)	3365 (67.4)	922 (65.3)
Smoking status (%)							
Never	17 356 (70.5)	2035 (71.1)	1098 (72.2)	6285 (72.2)	3626 (71)	3395 (68)	917 (65)
Former	2880 (11.7)	227 (7.9)	154 (10.1)	967 (11.1)	615 (12)	666 (13.3)	251 (17.8)
Current	4370 (17.8)	600 (21)	269 (17.7)	1455 (16.7)	869 (17)	934 (18.7)	243 (17.2)
Current drinking (%)							
Never	18 065 (73.4)	2187 (76.4)	1152 (75.7)	6555 (75.3)	3761 (73.6)	3515 (70.4)	895 (63.4)
Former	1400 (5.7)	130 (4.5)	68 (4.5)	496 (5.7)	279 (5.5)	308 (6.2)	119 (8.4)
Current	5141 (20.9)	545 (19)	301 (19.8)	1656 (19)	1070 (20.9)	1172 (23.5)	397 (28.1)

Abbreviations: MET, metabolic equivalent; BMI, body mass index.

Data were presented as number (%).

^*^Lower education was defined as junior high school or lower.

**Table 2:** Number of death (mortality rates in 1000 person-years) by regular physical activity among study participants.

Cause of death*	Overall	Regular physical activity in MET-hours/week					
		0	0.1 to <7.5	7.5 to <22.5	22.5 to <37.5	37.5 to <75.0	≥75
Total	1267 (10.1)	229 (1.8)	91 (0.7)	477 (3.8)	217 (1.7)	201 (1.6)	52 (0.4)
Neoplasms	436 (3.5)	56 (0.4)	33 (0.3)	162 (1.3)	88 (0.7)	79 (0.6)	18 (0.1)
Lung cancer	150 (1.2)	23 (0.2)	12 (0.1)	55 (0.4)	31 (0.2)	22 (0.2)	7 (0.1)
Liver cancer	53 (0.4)	5 (0.0)	5 (0.0)	17 (0.1)	12 (0.1)	13 (0.1)	1 (0.0)
Colon and rectum	45 (0.4)	7 (0.1)	4 (0.0)	18 (0.1)	9 (0.1)	6 (0.0)	1 (0.0)
Diseases of the circulatory system	458 (3.6)	99 (0.8)	37 (0.3)	171 (1.4)	72 (0.6)	61 (0.5)	18 (0.1)
Ischemic heart diseases	203 (1.6)	41 (0.3)	14 (0.1)	80 (0.6)	35 (0.3)	25 (0.2)	8 (0.1)
Cerebrovascular diseases	199 (1.6)	43 (0.3)	16 (0.1)	74 (0.6)	29 (0.2)	28 (0.2)	9 (0.1)
Diseases of the respiratory system	84 (0.7)	16 (0.1)	7 (0.1)	33 (0.3)	16 (0.1)	9 (0.1)	3 (0.0)
Diseases of the digestive system	37 (0.3)	5 (0.0)	2 (0.0)	17 (0.1)	5 (0.0)	5 (0.0)	3 (0.0)
External causes of morbidity and mortality	46 (0.4)	8 (0.1)	2 (0.0)	15 (0.1)	6 (0.0)	9 (0.1)	6 (0.0)

Abbreviations: MET, metabolic equivalent.

^*^The causes of death were coded according to the 10^th^ version of the International Classification of Diseases (ICD-10).

**Table 3:** Estimated hazard ratios (95% CIs) of total and cause-specific mortality associated with regular physical activity in MET-hours/week.

Cause of death*	>0 vs 0 MET-hours/week	Each SD increase	Regular physical activity in MET-hours/week					*P* linear trend
			0.1 to <7.5	7.5 to <22.5	22.5 to <37.5	37.5 to <75.0	≥75	
Total	0.54 (0.47–0.63)	0.78 (0.73–0.83)	0.69 (0.54–0.88)	0.61 (0.52–0.72)	0.53 (0.44–0.64)	0.43 (0.35–0.52)	0.39 (0.29–0.53)	<0.001
Neoplasms	0.80 (0.61–1.07)	0.88 (0.79–0.97)	1.03 (0.67–1.58)	0.86 (0.63–1.16)	0.86 (0.61–1.20)	0.68 (0.48–0.96)	0.53 (0.31–0.90)	0.009
Lung cancer	0.66 (0.42–1.04)	0.84 (0.71–1.00)	0.88 (0.44–1.78)	0.73 (0.45–1.20)	0.74 (0.43–1.27)	0.47 (0.26–0.84)	0.50 (0.21–1.17)	0.049
Diseases of the circulatory system	0.44 (0.35–0.55)	0.70 (0.62–0.78)	0.65 (0.44–0.95)	0.51 (0.40–0.65)	0.42 (0.31–0.57)	0.31 (0.22–0.42)	0.32 (0.19–0.53)	<0.001
Ischemic heart diseases	0.49 (0.35–0.70)	0.72 (0.61–0.85)	0.59 (0.32–1.09)	0.59 (0.40–0.86)	0.51 (0.32–0.80)	0.32 (0.19–0.52)	0.36 (0.17–0.77)	<0.001
Cerebrovascular diseases	0.43 (0.31–0.61)	0.71 (0.60–0.85)	0.64 (0.36–1.15)	0.50 (0.34–0.73)	0.39 (0.24–0.62)	0.32 (0.20–0.52)	0.37 (0.18–0.76)	<0.001
Diseases of the respiratory system	0.51 (0.30–0.89)	0.68 (0.52–0.90)	0.73 (0.30–1.77)	0.60 (0.33–1.10)	0.57 (0.29–1.15)	0.28 (0.12–0.63)	0.34 (0.10–1.19)	0.007

Abbreviations: MET, metabolic equivalent. SD, standard deviation.

Hazard ratios were estimated by using the Cox proportional hazards model, with adjustment for sex, age, BMI, marriage, education, smoking status and drinking status at baseline.

^*^The causes of death were coded according to the 10^th^ version of the International Classification of Diseases (ICD-10).

**Table 4:** Estimated hazard ratios (95% CIs) of total mortality associated with regular physical activity stratified by selected characteristics.

Characteristic	Regular vs irregular	Each SD increase	Regular physical activity in MET-hours/week					*P* trend	*P* effect modification
			0.1 to <7.5	7.5 to <22.5	22.5 to <37.5	37.5 to <75.0	≥75		
Sex									0.25
Male	0.57 (0.47–0.69)	0.84 (0.78–0.90)	0.64 (0.46–0.90)	0.63 (0.51–0.78)	0.58 (0.46–0.74)	0.48 (0.38–0.61)	0.44 (0.31–0.63)	<0.001	
Female	0.51 (0.41–0.64)	0.67 (0.59–0.76)	0.71 (0.50–1.02)	0.59 (0.46–0.75)	0.46 (0.33–0.62)	0.35 (0.25–0.48)	0.30 (0.16–0.56)	<0.001	
Age, years									0.08
≤60	0.50 (0.34–0.75)	0.81 (0.67–0.97)	0.24 (0.08–0.67)	0.64 (0.41–0.99)	0.43 (0.25–0.74)	0.49 (0.29–0.83)	0.36 (0.14–0.93)	0.025	
60–70	0.65 (0.50–0.83)	0.84 (0.76–0.92)	0.91 (0.61–1.36)	0.71 (0.54–0.93)	0.66 (0.49–0.89)	0.52 (0.39–0.71)	0.51 (0.33–0.79)	<0.001	
>70	0.49 (0.40–0.60)	0.72 (0.65–0.80)	0.66 (0.48–0.92)	0.55 (0.44–0.69)	0.48 (0.37–0.63)	0.36 (0.27–0.48)	0.32 (0.20–0.53)	<0.001	
BMI, kg/m^2^									0.99
<18.5	0.63 (0.34–1.14)	0.83 (0.64–1.08)	0.54 (0.20–1.47)	0.80 (0.42–1.53)	0.62 (0.29–1.33)	0.39 (0.17–0.90)	0.68 (0.19–2.47)	0.169	
18.5–24.9	0.58 (0.47–0.72)	0.77 (0.71–0.84)	0.73 (0.52–1.03)	0.67 (0.53–0.84)	0.60 (0.46–0.78)	0.45 (0.34–0.59)	0.37 (0.24–0.57)	<0.001	
25–27.9	0.50 (0.38–0.66)	0.81 (0.72–0.92)	0.61 (0.37–1.01)	0.56 (0.41–0.76)	0.45 (0.31–0.66)	0.45 (0.32–0.66)	0.34 (0.18–0.62)	<0.001	
≥28	0.58 (0.40–0.85)	0.78 (0.65–0.95)	0.75 (0.38–1.49)	0.64 (0.42–0.96)	0.51 (0.30–0.87)	0.45 (0.26–0.76)	0.63 (0.29–1.38)	0.012	
Marriage									0.64
Unmarried or divorced	0.65 (0.44–0.94)	0.74 (0.63–0.89)	0.96 (0.55–1.69)	0.76 (0.51–1.14)	0.51 (0.30–0.84)	0.50 (0.30–0.81)	0.51 (0.22–1.16)	<0.001	
Married	0.52 (0.45–0.61)	0.78 (0.73–0.84)	0.63 (0.48–0.83)	0.59 (0.49–0.70)	0.53 (0.43–0.65)	0.42 (0.34–0.51)	0.37 (0.27–0.51)	<0.001	
Education									0.81
Junior high school or lower	0.54 (0.40–0.73)	0.80 (0.71–0.91)	0.74 (0.46–1.19)	0.60 (0.43–0.83)	0.50 (0.34–0.74)	0.47 (0.32–0.69)	0.35 (0.19–0.64)	<0.001	
Senior high school or higher	0.55 (0.47–0.66)	0.78 (0.72–0.84)	0.67 (0.50–0.90)	0.63 (0.52–0.76)	0.56 (0.45–0.69)	0.43 (0.34–0.53)	0.42 (0.30–0.60)	<0.001	
Smoking status									0.04
Never	0.50 (0.41–0.60)	0.67 (0.61–0.74)	0.68 (0.50–0.94)	0.58 (0.47–0.72)	0.50 (0.39–0.65)	0.35 (0.27–0.45)	0.24 (0.15–0.41)	<0.001	
Current or former	0.61 (0.49–0.76)	0.89 (0.82–0.96)	0.69 (0.47–1.00)	0.66 (0.51–0.84)	0.58 (0.44–0.77)	0.55 (0.41–0.72)	0.56 (0.38–0.83)	0.005	
Drinking status									0.39
Never	0.54 (0.46–0.64)	0.73 (0.67–0.79)	0.70 (0.53–0.94)	0.63 (0.53–0.77)	0.50 (0.40–0.63)	0.41 (0.33–0.52)	0.29 (0.18–0.45)	<0.001	
Current or former	0.56 (0.43–0.73)	0.88 (0.80–0.97)	0.65 (0.41–1.02)	0.58 (0.43–0.78)	0.61 (0.44–0.85)	0.47 (0.34–0.66)	0.58 (0.37–0.89)	0.008	

Abbreviations: BMI, body mass index; MET, metabolic equivalent. SD, standard deviation.

Hazard ratios were estimated by using the Cox proportional hazards model, with adjustment for sex, age, BMI, marriage, education, smoking status and drinking status at baseline.
